# Aggregation of Halloysite Nanotubes in the Presence of Multivalent Ions and Ionic Liquids

**DOI:** 10.1021/acs.langmuir.1c01949

**Published:** 2021-10-04

**Authors:** Bojana Katana, Dóra Takács, Adél Szerlauth, Szilárd Sáringer, Gábor Varga, Andrej Jamnik, Felix D. Bobbink, Paul J. Dyson, Istvan Szilagyi

**Affiliations:** † MTA-SZTE Lendület Biocolloids Research Group, Interdisciplinary Excellence Center, Department of Physical Chemistry and Materials Science, University of Szeged, H-6720 Szeged, Hungary; ‡ Material and Solution Structure Research Group, Department of Organic Chemistry, University of Szeged, H-6720 Szeged, Hungary; § Faculty of Chemistry and Chemical Technology, University of Ljubljana, Večna pot 113, SI-1000 Ljubljana, Slovenia; ∥ Institute of Chemical Sciences and Engineering, École Polytechnique Fédérale de Lausanne (EPFL), CH-1015 Lausanne, Switzerland

## Abstract

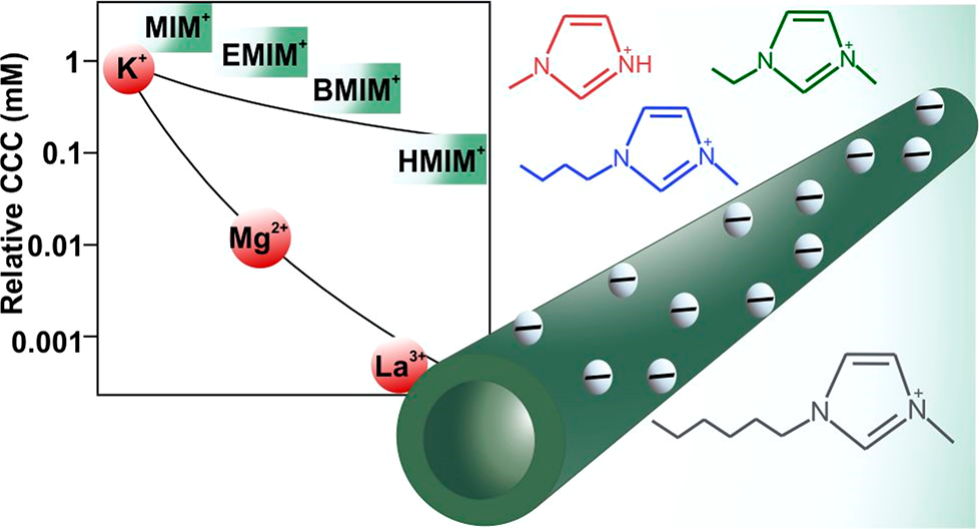

Colloidal stability was investigated in two types of particle systems, namely, with bare (h-HNT) and polyimidazolium-functionalized (h-HNT–IP-2) alkali-treated halloysite nanotubes in solutions of metal salts and ionic liquids (ILs). The valence of the metal ions and the number of carbon atoms in the hydrocarbon chain of the IL cations (1-methylimidazolium (MIM^+^), 1-ethyl-3-methylimidazolium (EMIM^+^), 1-butyl-3-methylimidazolium (BMIM^+^), and 1-hexyl-3-methylimidazolium (HMIM^+^)) were altered in the measurements. For the bare h-HNT with a negative surface charge, multivalent counterions destabilized the dispersions at low values of critical coagulation concentration (CCC) in line with the Schulze–Hardy rule. In the presence of ILs, significant adsorption of HMIM^+^ took place on the h-HNT surface, leading to charge neutralization and overcharging at appropriate concentrations. A weaker affinity was observed for MIM^+^, EMIM^+^, and BMIM^+^, while they adsorbed on the particles to different extents. The order HMIM^+^ < BMIM^+^ < EMIM^+^ < MIM^+^ was obtained for the CCCs of h-HNT, indicating that HMIM^+^ was the most effective in the destabilization of the colloids. For h-HNT–IP-2 with a positive surface charge, no specific interaction was observed between the salt and the IL constituent cations and the particles, i.e., the determined charge and aggregation parameters were the same within experimental error, irrespective of the type of co-ions. These results clearly indicate the relevance of ion adsorption in the colloidal stability of the nanotubes and thus provide useful information for further design of processable h-HNT dispersions.

## Introduction

Halloysite nanotubes (HNT)^1−3^ are layered aluminosilicates (Al_2_Si_2_O_5_(OH)_4_·*n*H_2_O) with hollow tubular structure possessing opposite signs of charges on the outer surface and inside the lumen.^4^ The outer surface is negatively charged in aqueous dispersions at intermediate pH values due to the presence of deprotonated silanol groups, whereas the inner lumen is positively charged due to the ionized aluminol groups.^5^ This dual charge nature combined with biocompatibility and large-scale accessibility make HNTs a promising candidate in a variety of applications, such as in biomedical and environmental sciences, wastewater treatment, nanoelectronics, and catalytic processes.^6−11^ HNTs have been extensively characterized in the solid state with microscopy, spectroscopy, and scattering techniques. The outer diameter of the tubes is about 40–70 nm, the inner diameter is 10–20 nm, and their length is typically 500–1500 nm.^12^ Unfortunately, the colloidal stability of commercially available HNTs is limited in aqueous environments, i.e., their dispersions can be easily destabilized by electrolyte-induced aggregation and subsequent sedimentation.^13,14^ However, alkaline treatment of HNTs (results in h-HNTs) increases the number of surface silanol groups, leading to improved colloidal stability.^15^ In addition, aggregation processes of HNTs (and h-HNTs) can be also tuned by surface modification with appropriate polyelectrolytes.^15−18^

Since HNT particles may be dispersed in liquid media, usually in aqueous electrolyte solutions for many applications, a comprehensive understanding of their colloidal stability is necessary prior to applying them in such media. In general, the aggregation features of dispersed particles in dielectric media such as salt solutions can be adequately described with the Derjaguin, Landau, Verwey, and Overbeek (DLVO) theory at different ionic strengths.^19,20^ It interprets the dispersion stability by considering van der Waals attractions between the particles and the repulsions as a result of the overlap of the electrical double layers. Stable dispersions are predicted at low electrolyte concentrations, i.e., at low ionic strengths, while particles tend to aggregate with increasing levels of dissolved salts. These two regimes (reported as slow and fast aggregation regimes) are typically separated by the critical coagulation concentration (CCC).^21^ The DLVO theory considers point-like charges in dielectric media; therefore, ions of the same valences are considered equally in the model, and thus, the same CCC is predicted irrespective of the type of ions present in the system. Nevertheless, many experimental results pointed out that the CCC changes once the chemical composition of the co-ions or counterions of the same valence is varied.^22−26^ This deviation from DLVO theory was often interpreted that ions of different chemical compositions adsorb to surfaces to a different extent due to specific interactions between the ion and the surface. This issue can be resolved with the Hofmeister series of ions, which orders the salt constituent ions by their power in destabilization of dispersions, while surface properties such as hydrophobicity and type of functional group should also be taken into account.^27−29^ However, for the multivalent ions, DLVO theory takes the valence of ions into account and predicts a decrease of the CCC with increasing valency through the Schulze–Hardy rule.^30−32^ Furthermore, the extent of this decrease is different for co-ions^33^ and counterions^34^ as well as depending on the magnitude of the surface charge.^35^ Hence, the ions of higher valences are more effective in destabilization of the colloidal dispersions. In this way, numerous studies have been published with systems containing nanoparticles and electrolytes of different valences and compositions, in which the aggregation mechanism and predominating interparticle forces were identified;^36−41^ nevertheless, very limited information is available in the literature for HNT materials in this respect.^13,14^

In addition to the aqueous media discussed above, novel solvents come into play in the applications of HNT materials. For instance, systems composed of HNT particles and ionic liquids (ILs) have attracted considerable contemporary interest in applications^42^ as anticorrosion agents,^43^ catalysts,^44^ or highly conductive materials.^45^ ILs are organic salts of low melting point and possess several favorable features including a wide electrochemical window, low vapor pressure, high chemical stability, and advantageous interfacial properties.^46−51^ Nanoparticles dispersed in ILs are widely used systems in various applications. However, the aggregation processes and relevant colloidal stability must be considered since stable dispersions containing well-dispersed particles are required in catalysis^52^ for instance, whereas controlled particle aggregation in ILs can be the basis of the preparation of novel materials.^53,54^ To explore the fundamental charging and aggregation features, the affinity of IL cations and anions to particle surfaces was studied. Findings with polystyrene,^23^ melamine,^55^ and silica^56^ particles shed light on the importance of ion-specific adsorption from aqueous IL solution, which governed the surface charge properties, while the slightly modified DLVO theory was able to describe the origin of the interparticle forces. The original Hofmeister series was also extended with IL cations and anions based on the nature of their interactions with various surfaces and macromolecules.^14,23,57,58^ Nevertheless, to the best of our knowledge, the colloidal stability of HNTs in IL solutions has been reported only once in the past.^14^

Therefore, in the present study, the charging and aggregation properties of bare and polyelectrolyte-coated h-HNT particles were studied in the presence of electrolytes of different valences and in aqueous solutions containing imidazolium-based ILs with different alkyl chain lengths. The influence of the electrolyte and IL cations on the colloidal stability was systematically studied, which were present in the samples either as co-ions or counterions.

## Experimental Methods

### Materials

ILs, 1-methylimidazolium chloride (MIMCl, >95%), 1-ethyl-3-methylimidazolium chloride (EMIMCl, >98%), 1-butyl-3-methylimidazolium chloride (BMIMCl, >99%), and 1-hexyl-3-methylimidazolium chloride (HMIMCl, >98%) were purchased from Iolitec GmbH. Analytical-grade salts such as potassium chloride (KCl) and magnesium chloride (MgCl_2_) were bought from VWR, and lanthanum chloride (LaCl_3_) was obtained from Alfa Aesar. The raw HNT powder was purchased from Sigma-Aldrich and subjected to alkaline activation treatment before use.^15^ For sample preparation, ultrapure water (resistivity of 18.2 mΩ·cm) was obtained from a VWR Purity TU+ instrument. The experiments were performed at 25 °C, and the h-HNT concentration was adjusted to 10 mg/L. The water, salt, and IL solutions were filtered with a 0.1 μm syringe filter (Millex). No unexpected or unusually high safety hazards were encountered.

The synthesis of the polyimidazolium-based polyelectrolyte (IP-2) has been reported earlier.^59^ Accordingly, a solution containing 1,4-bis(chloromethyl)benzene and 1-(trimethylsilyl)imidazole in a 1:1 molar ratio was prepared in acetonitrile in a Schlenk flask, and it was refluxed for 48 h. The white-colored solid product was filtered, washed with acetonitrile and diethyl ether, and dried under vacuum for 24 h. The chemicals used for the above synthetic process were purchased from Sigma-Aldrich.

The IP-2-modified h-HNT stock dispersion (denoted as h-HNT–IP-2 thereafter) was prepared by simply mixing calculated volumes of a 1 g/L IP-2 solution and 10 g/L h-HNT dispersion followed by appropriate dilution with water. The h-HNT–IP-2 contained 200 mg of IP-2 per 1 g of h-HNT. This preparation was carried out at neutral pH; however, the charge balance remains the same in the pH range 3–7, so the above protocol can be applied at different pHs too.

### Raman Spectroscopy

Raman measurements were performed with a Bruker Senterra II Raman microscope at an excitation wavelength of 765 nm applying 100 mW laser power and averaging 128 spectra with an exposition time of 6 s.

### Electron Microscopy

Morphology studies of bare and IP-2-modified h-HNTs were carried out using transmission electron microscopy (TEM) with a FEI Tecnai G^2^-20 X-Twin microscope. The h-HNT and h-HNT–IP-2 dispersions of 10 mg/L concentration were dried on copper mesh grids with carbon support (CF200-Cu, Electron Microscopy Sciences), and the images in bright-field mode were obtained at a 200 kV acceleration voltage using a LaB6 cathode.

### Electrophoresis

The electrophoretic light-scattering measurements were performed on a Litesizer 500 device (Anton Paar) using a 40 mW semiconductor laser operating at a 658 nm wavelength. The samples were prepared via the following procedure. A 1.8 mL amount of IL solutions of various concentrations was mixed with 0.2 mL of h-HNT dispersion of 100 mg/L particle dose. The samples were allowed to rest for 2 h at room temperature before measuring the electrophoretic mobilities, which occurred after a 1 min equilibration time in the device. The measurements were performed in 700 μL omega-shaped plastic cuvettes (Anton Paar). The reported data are the average of the results of five individual measurements. In all cases, the obtained electrophoretic mobility (*u*) data were converted to zeta potentials (ζ) with the Smoluchowski equation^60^
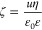
1where η is the dynamic viscosity, ε is the dielectric constant of the medium, and ε_0_ is the permittivity of the vacuum. The charge density of the particles (σ) at the slip plane was determined with the Debye–Hückel model^61^
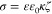
2where κ is the inverse Debye length.^60^

### Dynamic Light Scattering

Dynamic light scattering (DLS) was used to measure the hydrodynamic radius (*R*_h_) of the particles. The experiments were carried out with the same Litesizer 500 instrument as used during electrophoresis at a scattering angle of 175°. The correlation function was fitted with the cumulant method to obtain the decay rate constant (Γ). The translational diffusion coefficient (*D*) was calculated as follows^62^
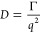
3where *q* is the scattering vector, which can be calculated using the parameters of the experimental setup as
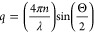
4where *n* is the refractive index of the medium, λ is the wavelength of the laser beam, and Θ is the scattering angle. *R*_h_ was then calculated by the Stokes–Einstein equation^63^
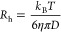
5where *k*_B_ is the Boltzmann constant and *T* is the temperature. The same sample preparation procedure was applied as in the electrophoretic measurements; however, the DLS experiments were started after adding the particle stock dispersion and mixing for 25 s. The samples were equilibrated for 30 s in the device prior to data collection. From time-resolved DLS experiments, the stability ratio (*W*) values were calculated as follows^62,64^
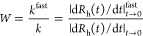
6where *k* is the apparent aggregation rate constant calculated from the increase in the *R*_h_ data and *k*^fast^ is the rate constant determined in 1 M KCl solutions, in which diffusion-controlled particle aggregation occurs. Hence, the stability ratio is one in the case of unstable samples, and higher values indicate slower particle aggregation. The transition between the fast and the slow aggregation regimes is located at the CCC, whose value was determined as follows^64^
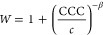
7where *c* is the molar concentration and β is calculated from the slope of the stability ratios before the CCC as
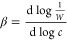
8

## Results and Discussion

First, the h-HNT particles were functionalized with IP-2 polymer, and both systems, bare and IP-2-modified h-HNTs, were characterized by different techniques to verify the successful adsorption of IP-2 on the h-HNT particles. In addition, the colloidal stability of both systems was studied in the presence of metal salts and ionic liquid constituents by light-scattering techniques. The experimental conditions were kept constant in both types of measurements.

### Functionalization of h-HNT with IP-2

**Figure 1:**
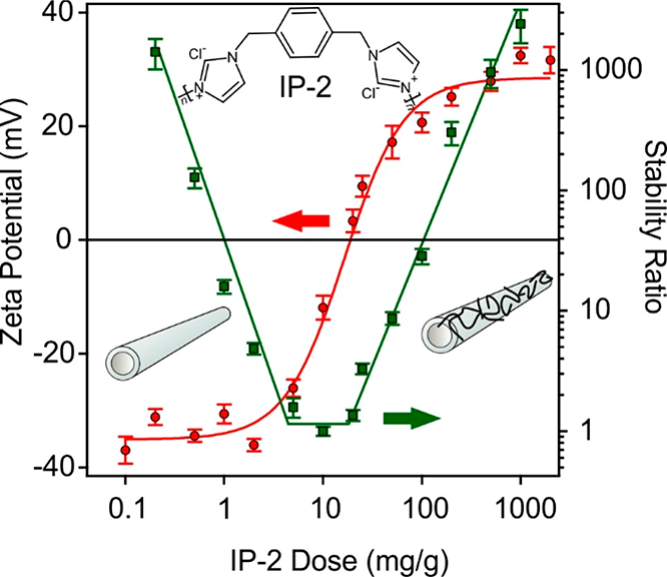
Zeta potentials (circles, left axis) and stability ratios (squares, right axis) of h-HNT particles versus the IP-2 dose at pH 7 and 10 mM ionic strength adjusted by KCl. Unit mg/g refers to milligrams of IP-2 per gram of h-HNT. Lines (red for the zeta potentials and green for the stability ratios) are eye guides. Structure presented at the top refers to IP-2. Two inset pictures represent the bare h-HNT (left) and h-HNT–IP-2 (right).

To obtain the h-HNT–IP-2 particles, h-HNT was functionalized with IP-2 through adsorption of the polyelectrolyte on the oppositely charged surface. Such an adsorption process was followed, and the IP-2 dose needed to coat the h-HNT was determined in the zeta potential measurements performed at different IP-2 doses. As shown in Figure 1, negative zeta potentials were recorded at low IP-2 loadings, and they increased as the amount of added polyelectrolyte was increased, indicating the adsorption process on the oppositely charged surface. Such adsorption led to charge neutralization and overcharging at appropriate polymer doses, similar to other systems containing polyelectrolytes and oppositely charged surfaces.^15,22,65^ At IP-2 doses higher than 200 mg/g, the zeta potentials remained constant due to the formation of a saturated IP-2 layer on the h-HNT surface, which provided a relatively high positive charge for the particles.

**Figure 2:**
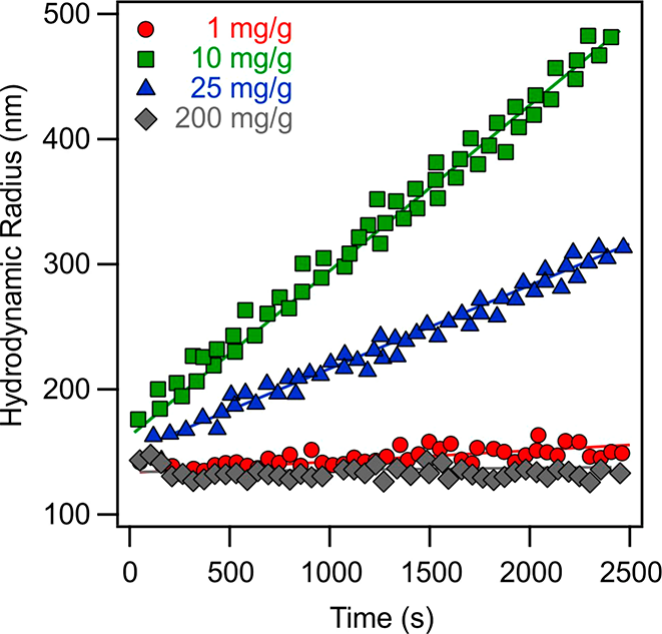
Hydrodynamic radii versus time at different doses of IP-2 measured in time-resolved DLS experiments. Experiments were performed at pH 7, 10 mM ionic strength adjusted by KCl and 10 mg/L h-HNT concentration. Unit mg/g refers to milligrams of IP-2 per gram of h-HNT, and lines are linear fits used for calculation of the stability ratios by eq 6.

Aggregation of the IP-2-modified h-HNT particles was investigated at different IP-2 doses by time-resolved DLS (Figure 2). The hydrodynamic radius changed slightly at an IP-2 dose of 1 mg/g, where the h-HNT surface is partially neutralized with IP-2. A more significant increase was observed at a dose of 25 mg/g, at which the particles are positively charged. The largest increase was recorded at 10 mg/g IP-2 loading. Comparing with the zeta potential data, these doses are located below, above, and at the charge neutralization point, respectively. In addition, no aggregation was detected at a 200 mg/g dose, where the zeta potential measurements indicate the formation of a saturated IP-2 layer on the h-HNT. The aggregation tendencies were further assessed by determining the stability ratio values under the same experimental conditions as those used for the electrophoretic measurements.

Accordingly, fast particle aggregation occurred near the charge neutralization point (Figure 1), while the stability ratios increased away from this point, indicating stabilization of the dispersions. Such a U-shaped curve is typical for systems containing oppositely charged polyelectrolytes and particles including HNTs.^15,17,22,66^ Referring to the data sets shown in Figure 2, stable samples were observed at 1 and 200 mg/g, moderate stability was observed at 25 mg/g, while rapid particle aggregation occurred at 10 mg/g. These findings are in qualitative agreement with the prediction of DLVO theory, since attractive forces destabilize the samples at charge neutralization (10 mg/g) while sufficiently charged particles (1 and 200 mg/g) are stable due to repulsion between the electrical double layers.

Considering the electrophoretic and DLS results, a dose of 200 mg/g was chosen for further measurements, because the h-HNT surface is completely coated with IP-2 and a stable dispersion is formed at this dose. Therefore, h-HNT–IP-2 refers to the composite of 200 mg/g polyelectrolyte content.

### Particle Characterization in Solid State

**Figure 3:**
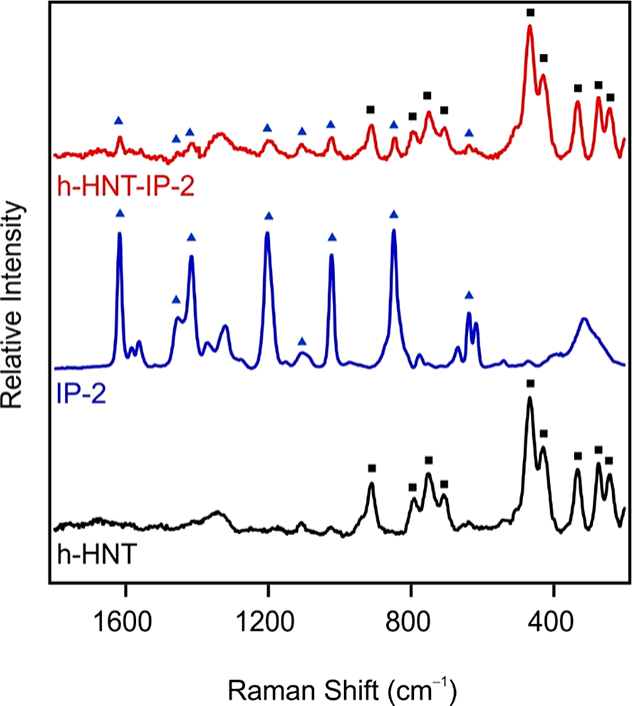
Raman spectra of h-HNT, IP-2, and h-HNT–IP-2. Characteristic vibrational bands of h-HNTs and IP-2 are indicated by squares and triangles, respectively.

To reveal the structural features of h-HNT–IP-2, its Raman spectrum was measured and compared to the spectra of h-HNTs and IP-2. The spectra are presented in Figure 3, and the full assignment of the Raman bands is listed in Table 1.

**Table 1:** Observed Raman Bands and Their Assignments to the Components of the h-HNT–IP-2 Hybrid Material^a^

wavenumber (cm^–1^)	assignment	compound
243	ν_sym_(M–OH)	h-HNT, h-HNT–IP-2
274	ν_asym_(M–OH)	h-HNT, h-HNT–IP-2
333	δ(M–OH···OH)	h-HNT, h-HNT–IP-2
430	ν(Al–O)	h-HNT, h-HNT–IP-2
468	δ(Si–O)	h-HNT, h-HNT–IP-2
708, 750, 792	Γ_(T)_(Al–OH)	h-HNT, h-HNT–IP-2
909	Γ_(T′)_(Al–OH)	h-HNT, h-HNT–IP-2
618,669	τ_ring_	IP-2
637	ρ_ring_	IP-2, h-HNT–IP-2
849	δ(N–C)	IP-2, h-HNT–IP-2
1023, 1105	δ_sym_(aromatic ring), δ_asym_(aromatic ring)	IP-2, h-HNT–IP-2
1203	ν(C^(+)^–N)	IP-2, h-HNT–IP-2
1321	ν_sym_(heteroaromatic ring)	IP-2
1416	ν_sym_(aromatic ring)	IP-2, h-HNT–IP-2
1455	ν_asym_(aromatic ring)	IP-2, h-HNT–IP-2
1563	ν_asym_(heteroaromatic ring)	IP-2
1583	ν_asym_(aromatic ring)	IP-2
1617	ν(C=N)	IP-2, h-HNT–IP-2

a ν = stretching vibration; sym = symmetric; asym = asymmetric; δ = bending vibration; Γ_(T)_ = translation mode; τ = twisting vibration; ρ = rocking vibration; M = metal.

The bands around 243 and 274 cm^–1^ are due to the symmetric and asymmetric stretching modes of the triangular M–OH group. The intense peak at 333 cm^–1^ is assigned to the H-bonded M–OH function. The other intense band at 468 cm^–1^ is related to the Si–O bending vibration. Raman bands with medium intensity associated with the Al–OH translation modes were detected at 708, 750, and 792 cm^–1^. The associated vibration mode of the inner Al–OH groups was observed as a single band at 909 cm^–1^.^67^

In the case of IP-2 polymer, characteristic peaks at 618, 637, and 669 cm^–1^ were identified as different deformation mode bands of the aromatic rings.^68^ In addition, further deformation bands of the aromatic rings were observed at 1023 and 1105 cm^–1^. Furthermore, additional stretching mode vibration bands of this organic moiety were observed in the region 1321–1583 cm^–1^. Besides, one of the most intense peaks at 849 cm^–1^ could be attributed to the N–C bending vibration.^69^ Furthermore, the band at 1203 cm^–1^ could be assigned to the C^(+)^–N vibration.^70^ The stretching mode vibration of the C=N moiety was also found at 1617 cm^–1^.

On the basis of the peak assignment, the presence of the polymer and particles was confirmed in h-HNT–IP-2. Note that the intensity of the IP-2 peaks decreased in the spectrum of the composite due to the higher sensitivity of the technique to h-HNT at the excitation wavelength used.^71,72^

**Figure 4:**
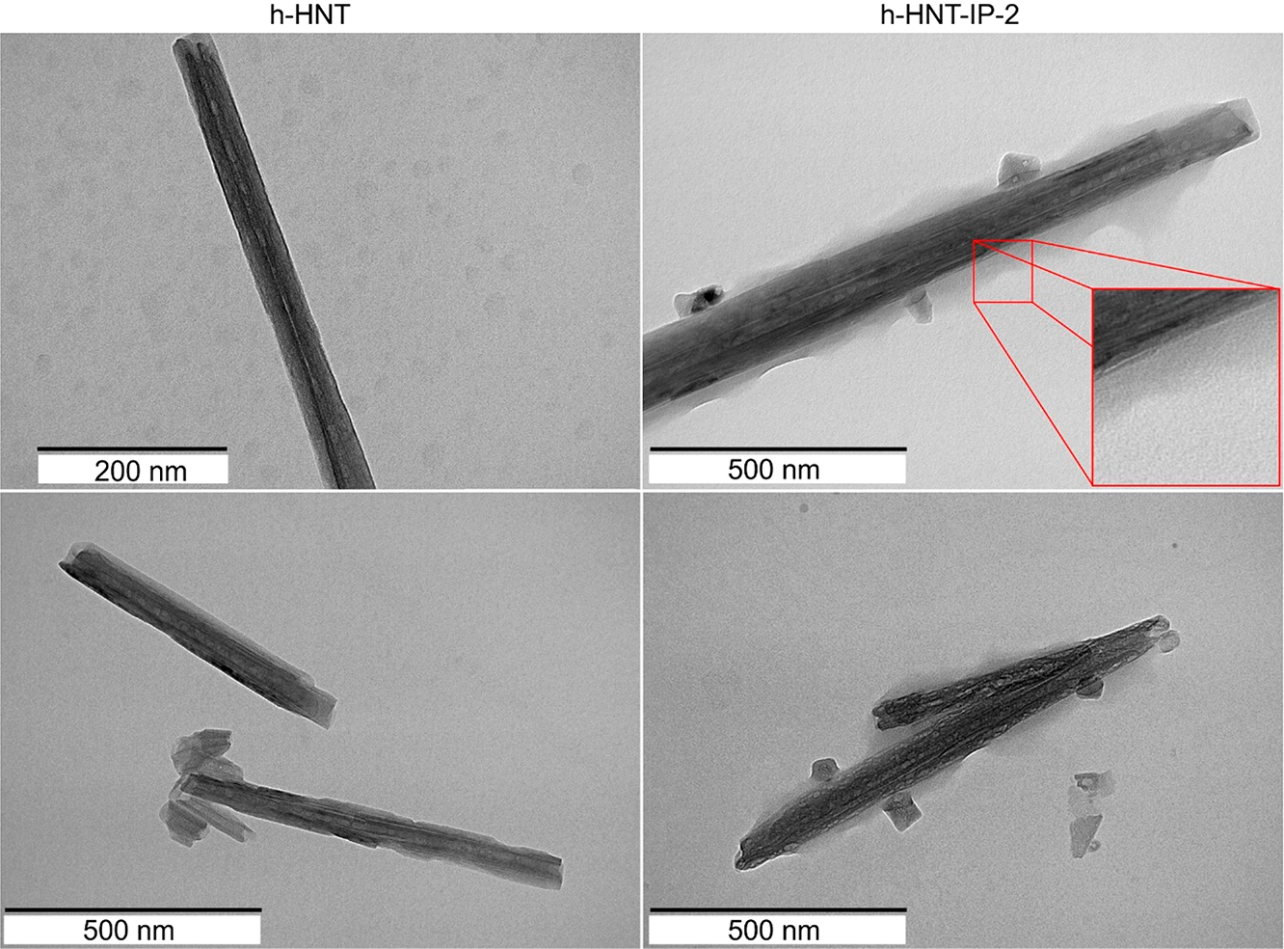
TEM images of the bare h-HNT (left column) and h-HNT–IP-2 (right column) materials. Images were taken after drying 10 mg/L dispersions on a copper–carbon mesh.

The morphology of bare h-HNTs and h-HNT–IP-2 was studied with TEM (Figure 4). The outer diameter and length of h-HNT and h-HNT–IP-2 vary in the range of 50–60 nm and 200–1500 nm, respectively, indicating reasonable polydispersity in these dimensions. The elongated structure of the particles was confirmed for both systems, i.e., the morphology of the nanotubes did not change upon polyelectrolyte adsorption. From the h-HNT–IP-2 images, a shadow-like thin layer around the particles was observed, which may correspond to the adsorbed IP-2 layer on the h-HNT surface.

### Colloidal Stability in the Presence of Monovalent and Multivalent Metal Ions

**Figure 5:**
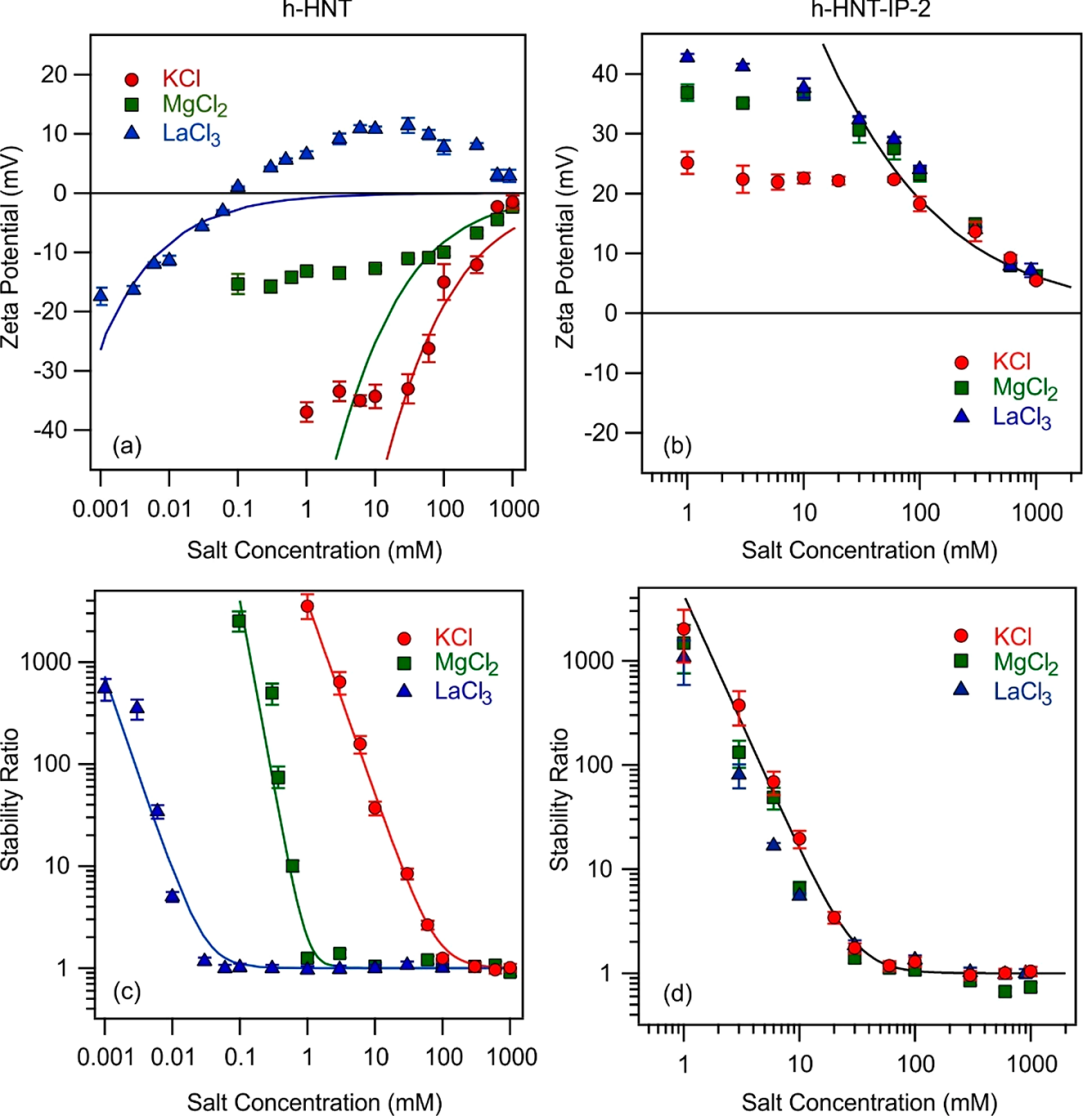
Zeta potentials (h-HNT (a) and h-HNT–IP-2 (b)) as well as stability ratios (h-HNTs (c) and h-HNT–IP-2 (d)) as a function of salt concentration at pH 7. Lines in a and b were calculated by eq 2 and in c and d by eq 7.

The charging and aggregation properties were investigated in the presence of monovalent (KCl) and multivalent (MgCl_2_ and LaCl_3_) salts. The zeta potentials measured for h-HNT and h-HNT–IP-2 are presented in Figure 5a and 5b, respectively.

For bare h-HNTs, the monovalent (K^+^) and multivalent (Mg^2+^ and La^3+^) ions are present as the counterions (for the h-HNT–IP-2 particles, they act as co-ions), i.e., they are of the same sign of charge as the surface. In general, zeta potential data were negative at low ionic strengths and increased with the salt concentrations in all systems. For K^+^ and Mg^2+^, the zeta potentials were close to zero at high-salt concentrations, while for La^3+^, adsorption of the trivalent cation caused charge neutralization and overcharging at sufficiently high concentrations. The data clearly show that the affinity of the cations to the surface increased by increasing the valence.

One of the most important parameters, which can be estimated from the electrolyte concentration dependence of the zeta potentials, is the charge density at the slip plane. This calculation is possible within the Debye–Hückel model, which is valid only for indifferent ions, whose adsorption on the surface is negligible.^61^ In addition, note that significant deviations between the experimental and the calculated zeta potential values occur at lower ionic strengths due to the electrokinetic effect.^73^ The calculated surface charge density values for the bare h-HNTs in the presence of K^+^, Mg^2+^, and La^3+^ are −1.4 × 10^–2^, −6.0 × 10^–3^, and −6.2 × 10^–5^ C/m^2^, respectively. Comparing the calculated and the experimental zeta potential data for the latter two, one can notice that these values are rather inaccurate, since the model described by eq 2 does not include specific ion adsorption, which can be clearly seen from the deviation between the measured and the calculated potential values.

On the other hand, for the h-HNT–IP-2 system, the zeta potential values were the same within the experimental error above 30 mM concentration, while differences were observed at lower salt concentrations (Figure 5b). With increasing electrolyte concentration, the zeta potential decreased after an intermediate small maximum due to the electrokinetic effect^73^ and remained positive in the entire concentration regime investigated. The obtained charge density values were the same within the measurement error (1.4 × 10^–2^ C/m^2^) irrespective of the type of metal salt, indicating the absence of specific adsorption of the co-ions. These results indicate that the adsorption of salt constituents on the h-HNT–IP-2 surface is negligible and that the decrease in the potentials with increasing ionic strength is due to electrostatic screening of the surface charge.

The stability ratios measured at different salt or IL concentrations are presented in Figure 5c and 5d, respectively. The general trends in the stability ratios were the same in all cases. They decreased with increasing the ionic strength and remained close to unity at high concentrations. Accordingly, slow aggregation was observed at low salt concentrations, as in this region the stability ratio values are high while the particles undergo rapid aggregation above the CCC. This behavior is in line with the qualitative prediction of DLVO theory,^20,74^ which states that the overlapping electrical double layers induce strong repulsion at low ionic strengths, giving rise to stabilized dispersions. Besides, at high salt concentrations, the extent of electrostatic repulsion decreases due to the screening effect of the salts and fast particle aggregation takes place owing to the presence of attractive forces. Such attractive forces originate from van der Waals interactions, and data of direct force measurements^75−77^ shed light on the fact that other non-DLVO attractive forces are also present at small surface separations. They result from ion correlations and surface heterogeneities.

For the monovalent salt, the stability ratio curve was identical within the experimental error with that reported earlier for the bare h-HNTs.^14^ The onset of the fast particle aggregation was located at the same CCC, namely, at 80 mM. The stability curves for the multivalent counterions contain the slow and fast aggregation regions, i.e., the tendencies in the data were like in the monovalent case. However, for the di- and trivalent ions, the CCCs were substantially lower compared to that determined for K^+^. The CCC for the divalent (Mg^2+^) counterion was found to be 1 mM, and for the trivalent (La^3+^) counterion it was located at 0.03 mM salt concentration. Therefore, the CCC data decrease with increasing valence, in good agreement with the Schulze–Hardy rule^31,78^ discussed later.

Nevertheless, the measured stability ratio values for the h-HNT–IP-2 dispersions were about the same for KCl, MgCl_2_, and LaCl_3_ (Figure 5d), and hence, an identical CCC value (30 mM) was determined for all systems. In general, a dependence of the CCC on the valence of the co-ions is predicted by the inverse Schulze–Hardy rule.^33,79^ However, there is no sign for such a decrease in the CCC by increasing the valency of the co-ions. This behavior indicates the powerful masking effects of the IP-2 polymer used for the functionalization of the h-HNT surface. In other words, the IP-2 coating gave rise to the formation of indifferent surfaces, which are insensitive for ion-specific effects. This conclusion is further confirmed by the very similar zeta potentials measured in the same systems. Such a masking phenomenon was already reported earlier in the case of polymeric latex particles functionalized with IP-2.^66^

### Colloidal Stability in IL Solutions

**Figure 6:**
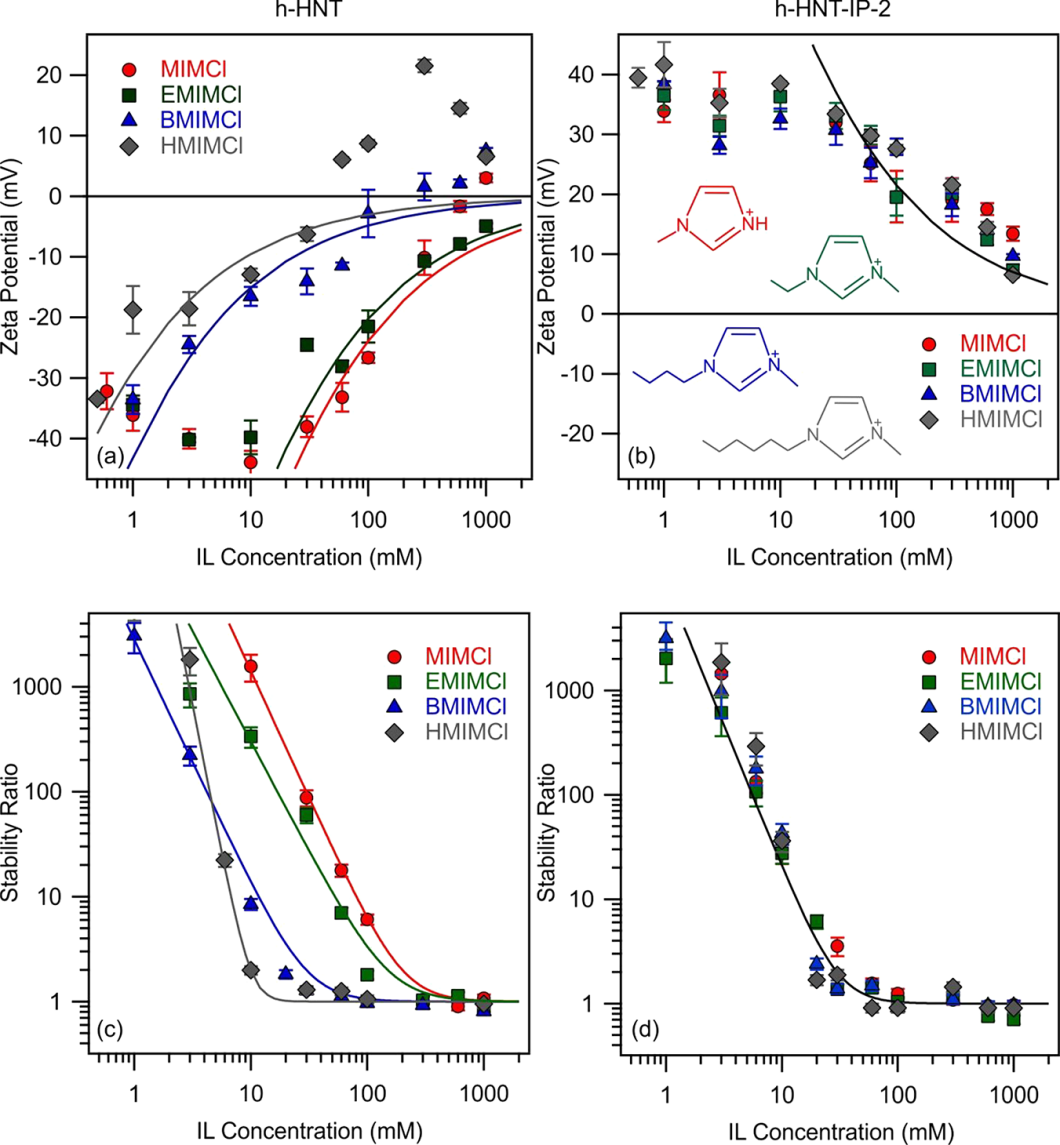
Zeta potentials (h-HNTs (a) and h-HNT–IP-2 (b)) as well as stability ratios (h-HNTs (c) and h-HNT–IP-2 (d)) as a function of IL concentration. Lines in a and b were calculated by eq 2 and in c and d by eq 7. Structure of IL cations is shown in the inset of b.

Zeta potentials were measured for bare h-HNT in the presence of MIMCl, EMIMCl, BMIMCl, and HMIMCl (Figure 6a) at different concentrations, the different IL cations being the counterions and chloride ions the co-ions. In all cases, the zeta potential increases with increasing the IL concentration. For MIM^+^, EMIM^+^, and BMIM^+^, the particles were negatively charged over the entire IL dose range studied. The increase is primarily owing to the screening effect of the ions by the IL constituents, but adsorption of ions of different affinities to the surface also leads to such an increase in the zeta potentials. Accordingly, the data indicate that BMIM^+^ has the highest affinity to the surface compared to MIM^+^ and EMIM^+^. Adsorption of the counterions becomes more pronounced for those with longer aliphatic chains, leading to a significant charge reversal for HMIM^+^. At higher concentrations, the zeta potentials decrease in this system due to charge screening. A similar behavior was reported earlier with these ILs^23^ as well as with aliphatic amines^80^ adsorbed on latex particles.

The surface charge density values were calculated for the h-HNTs in the presence of MIM^+^, EMIM^+^, BMIM^+^, and HMIM^+^, and they were estimated to be −1.8 × 10^–2^, −1.5 × 10^–2^, −4.0 × 10^–3^, and −2.0 × 10^–3^ C/m^2^, respectively. However, the striking deviation between the calculated and the measured zeta potential data for BMIM^+^ and HMIM^+^ indicates that the use of the Debye–Hückel model is not appropriate in these cases due to the strong counterion adsorption on the surface. For the h-HNT–IP-2 system, the addition of different IL co-ions (MIM^+^, EMIM^+^, BMIM^+^, and HMIM^+^) led to the same zeta potentials within the experimental error (Figure 6b), similar to results with h-HNT in salt solutions discussed above. Moreover, after an initial maximum,^73^ the zeta potential data decreased with increasing level of ILs for all systems, and the very similar potentials indicated that no specific adsorption of the IL cations occurred.

The stability ratios were determined for bare h-HNT in solutions of MIMCl, EMIMCl, BMIMCl, and HMIMCl (Figure 6c). The experimental conditions (e.g., pH, particle loading, and IL concentration range) were the same as those used in the zeta potential study in order to correlate the results. The bare h-HNT particles aggregated in a similar fashion in the IL solutions, and they followed DLVO-type behavior, namely, slow aggregation at low IL concentrations and rapid aggregation after the CCC. The only difference in the tendency of the stability ratios was observed for the HMIM^+^, where the slope in the slow aggregation regime was appreciably higher than that for the other systems. It was assumed that this is due to the strong adsorption of HMIM^+^ giving rise to somewhat different surface properties compared to the other systems, where counterion adsorption takes place to a smaller extent. Although the trend in the stability ratios was qualitatively in line with DLVO theory, the CCC values were different, which is in contrast with this model since DLVO theory predicts the same CCC for all monovalent salts irrespective of the type of ions. The CCC in the presence of the MIM^+^ was 200 mM IL and for EMIM^+^ was 150 mM. In addition, in the presence of BMIM^+^ and HMIM^+^ cations, the CCCs were found to be 30 and 10 mM, respectively. The CCC values followed the MIM^+^ > EMIM^+^ > BMIM^+^ > HMIM^+^ order, in accordance with the trend in the zeta potential data discussed above.

Once the number of carbon atoms was increased in the hydrocarbon chains of IL cations, no differences were detected in the colloidal behavior of the h-HNT–IP-2 particles. The measured stability ratio values were identical within experimental error for MIMCl, EMIMCl, BMIMCl, and HMIMCl (Figure 6d), and thus, the onset of the rapid particle aggregation is located at the same CCC of 30 mM IL concentration for all systems. Considering that the IL cations are the co-ions in these dispersions, one can conclude that the ILs simply destabilize h-HNT–IP-2 by charge screening and no specific interaction took place between the ILs and the IP-2-modified h-HNTs. As in the presence of inorganic salts discussed above and in line with results reported earlier,^66^ IP-2 functionalization prevented any ion-specific effects on the colloidal stability of the particles.

### CCC Dependence on the Valence and Alkyl Chain Length

**Figure 7:**
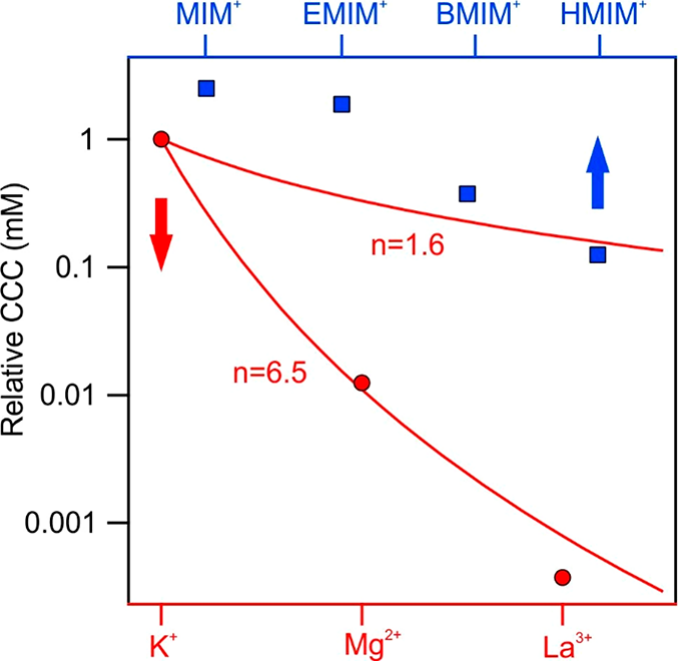
Relative CCC values (normalized to the CCC obtained in the presence of KCl) of bare h-HNTs for cations of either different valences (circles) or different alkyl chain lengths (squares). Solid lines indicate the direct (for *n* = 1.6 and 6.5 in eq 9) Schulze–Hardy rule.

The dispersion destabilizing power of the ionic valence was derived from the DLVO-based Schulze–Hardy rule, which indicates that the CCC decreases with the valence (*z*) of dissolved ions as follows^61,78^
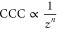
9where *n* changes between 1.6 and 6.5. This value is determined by the surface charge and related hydrophobicity of the particles. Accordingly, the exponent for weakly charged particles is *n* = 16 in eq 9, while for particles of high surface charge, the dependence is *n* = 6.5 if one considers asymmetric electrolytes.^35^ However, the latter number can only be derived from DLVO theory for surfaces with extremely high magnitude of charge, around 1 C/m^2^, while the surface charge density for inorganic particles, such as HNTs, is typically much less than this value. Figure 7 shows the relative CCCs normalized to the CCC obtained for KCl and the CCC values expected from the Schulze–Hardy rule (eq 9) with the above-mentioned limits.

The CCCs decreased with increasing valence in the order K^+^ > Mg^2+^ > La^3+^, as expected, and the decrease shows good quantitative agreement with the *n* = 6.5 dependence in eq 9. However, as discussed above, such a strong dependence can only be derived from DLVO theory for highly charged surfaces (above 1 C/m^2^ charge density), while the magnitude of the surface charge density determined for h-HNTs in KCl solutions was much smaller (1.4 × 10^–2^ C/m^2^ for KCl, for instance). It was assumed that the affinity of the cations, i.e., the strength of their adsorption to the oppositely charged h-HNTs, plays a major role in the aggregation processes. This is why multivalent ions adsorb to a larger extent and reduce the surface charge more significantly. This is in line with the results of the zeta potential measurements, as illustrated in Figure 5a. Once the surface charge density is reduced due to adsorption of counterions, a lower salt concentration is needed to destabilize the colloids and thus the CCC is lower. Therefore, the *n* = 6.5 dependence of the CCC on the valence is the common result of ion adsorption and prediction of DLVO theory, as they are very sensitive to the valence of counterions.

The relative CCCs determined in the presence of IL cations are also shown in Figure 7. Concerning the tendency, the values decrease in the sequence MIMCl > EMIMCl > BMIMCl > HMIMCl. This trend reflects that the interfacial processes are tuned by the alkyl chain length.^24^ Indeed, more hydrophobic cations of longer alkyl chains are located on the right-hand side, and they adsorb stronger to the particles, leading to lower CCCs, while the ones on the left-hand side are of more hydrophilic character due to shorter hydrocarbon chains. These counterions adsorb more weakly, giving rise to higher CCCs. The surface charge–CCC relation can be explained in a similar way to that discussed above with the multivalent ions. The above tendencies in the CCC are in good agreement with the findings reported earlier with colloidal particles dispersed in different metal salt or IL solutions.^34,36,37,41,80^

Another interesting finding, which deserves further discussion, is the higher CCCs for MIM^+^ and EMIM^+^ compared to that determined for KCl (Figure 7). It is suspected that this phenomenon is due to ion pair formation of ILs both on the surface and in the bulk. The latter has been already reported earlier,^51^ and such an association of the IL constituents in aqueous solution decreases the ionic strength. Thus, the repulsion between electrical double layers become stronger, leading to a higher CCC. On the other hand, adsorbed IL cations may attract chloride anions from the liquid phase and form ion pairs on the surface. This process results in a weaker charge neutralization, i.e., the surface charge is more negative, and it gives rise to a higher CCC than in the case of completely dissociated KCl. Once the cation adsorption is more pronounced, such as the BMIM^+^ and HMIM^+^, the influence of ion pair formation on the surface charges and corresponding interparticle forces becomes negligible.

## Conclusions

The charging and aggregation properties of clay nanotubes, i.e., bare (h-HNTs) and polyimidazolium-functionalized (h-HNT–IP-2), were investigated in the presence of metal salt and IL solutions. In the case of h-HNTs, increasing the valence and alkyl chain length of the IL cations increased the affinity of the counterions to the surface, as revealed by zeta potential measurements, and subsequently affected the surface charge density of the particles. Colloidal stability studies revealed that the particle aggregation mechanism in each system qualitatively followed the prediction of DLVO theory irrespective of the type of counterions. However, significant differences were observed in the obtained CCC values, whose order was determined as MIM^+^ > EMIM^+^ > K^+^ > BMIM^+^ > HMIM^+^ > Mg^2+^ > La^3+^. The sequence agrees adequately with the Schulze–Hardy rule for the metal ions as a joint result of specific ion adsorption and valence dependence of the electrical double layer forces predicted by DLVO theory. The trend in the CCCs for IL cations originates from the hydrophobic nature of the counterions, which became more pronounced with increasing alkyl chain length on the imidazolium ring. Accordingly, the HMIM^+^ cations exhibited the greatest affinity to the particle surface, giving rise to charge neutralization and overcharging, while such a significant adsorption gave rise to weaker surface charge and consequently to the lowest CCC within the IL cations. The MIM^+^ adsorption was the weakest, and thus, the highest CCC was determined for this IL constituent.

For the h-HNT–IP-2 particles, where the metal and IL cations were the co-ions, ion-specific effects were not observed in either salt or IL solutions. In other words, both the zeta potentials and the stability ratios were the same within the experimental error in the presence of different metal salts and ILs. Therefore, the same surface charge density was determined in the presence of the mono- and multivalent salt constituents as well as the IL cations. On the other hand, the aggregation mechanism was realized within DLVO theory in all systems investigated. These results are consistent with the previously reported conclusions that the saturated IP-2 layer on the particles masks ion-specific effects and the colloidal stability of h-HNT–IP-2 depends only on the ionic strength applied in the dispersions.

Given the growing interest in HNT materials suspended in electrolyte or IL solutions, the present results provide important quantitative information on the interfacial properties and subsequently on the aggregation of the particles. Such a comprehensive study on the colloidal stability of HNT particles for various salts and ILs has not been reported so far. Therefore, the results are expected to be used in the design of fine HNT dispersions in which the particle aggregation can be tuned by the ionic valence or by the length of the hydrocarbon chain of the IL constituent ions.
